# Complex-Morphology Metal-Based Nanostructures: Fabrication, Characterization, and Applications

**DOI:** 10.3390/nano6060110

**Published:** 2016-06-06

**Authors:** Antonella Gentile, Francesco Ruffino, Maria Grazia Grimaldi

**Affiliations:** 1 Department of Physics and Astronomy-University of Catania, via S. Sofia 64, 95123 Catania, Italy; antonella.gentile@ct.infn.it (A.G.); mariagrazia.grimaldi@ct.infn.it (M.G.G.); 2 MATIS IMM-CNR, via S. Sofia 64, 95123 Catania, Italy

**Keywords:** Au, Ag, nano-dendrites, peapodded nanowires, nanorices, nanorings, nanoporous Au, optical properties, plasmonics, SERS

## Abstract

Due to their peculiar qualities, metal-based nanostructures have been extensively used in applications such as catalysis, electronics, photography, and information storage, among others. New applications for metals in areas such as photonics, sensing, imaging, and medicine are also being developed. Significantly, most of these applications require the use of metals in the form of nanostructures with specific controlled properties. The properties of nanoscale metals are determined by a set of physical parameters that include size, shape, composition, and structure. In recent years, many research fields have focused on the synthesis of nanoscale-sized metallic materials with complex shape and composition in order to optimize the optical and electrical response of devices containing metallic nanostructures. The present paper aims to overview the most recent results—in terms of fabrication methodologies, characterization of the physico-chemical properties and applications—of complex-morphology metal-based nanostructures. The paper strongly focuses on the correlation between the complex morphology and the structures’ properties, showing how the morphological complexity (and its nanoscale control) can often give access to a wide range of innovative properties exploitable for innovative functional device production. We begin with an overview of the basic concepts on the correlation between structural and optical parameters of nanoscale metallic materials with complex shape and composition, and the possible solutions offered by nanotechnology in a large range of applications (catalysis, electronics, photonics, sensing). The aim is to assess the state of the art, and then show the innovative contributions that can be proposed in this research field. We subsequently report on innovative, versatile and low-cost synthesis techniques, suitable for providing a good control on the size, surface density, composition and geometry of the metallic nanostructures. The main purpose of this study is the fabrication of functional nanoscale-sized materials, whose properties can be tailored (in a wide range) simply by controlling the structural characteristics. The modulation of the structural parameters is required to tune the plasmonic properties of the nanostructures for applications such as biosensors, opto-electronic or photovoltaic devices and surface-enhanced Raman scattering (SERS) substrates. The structural characterization of the obtained nanoscale materials is employed in order to define how the synthesis parameters affect the structural characteristics of the resulting metallic nanostructures. Then, macroscopic measurements are used to probe their electrical and optical properties. Phenomenological growth models are drafted to explain the processes involved in the growth and evolution of such composite systems. After the synthesis and characterization of the metallic nanostructures, we study the effects of the incorporation of the complex morphologies on the optical and electrical responses of each specific device.

## 1. Introduction

Nanoscale-sized materials are materials with at least one dimension between 1 and 100 nm [1]. The crystalline nanomaterials are also characterized by a single-domain crystalline lattice, without the complicating presence of grain boundaries [2]. Scientific interest in nanomaterials has been growing steadily due to their unique position as a bridge between atoms and bulk solids, as well as their fascinating properties and potential applications [3]. The ability to generate such nanoscale-sized materials is central to advances in many areas of modern science and technology. In principle, the electron confinement by a nano-material provides the most powerful means to manipulate the electronic, optical, and magnetic properties of a solid material. This notion explains why nanomaterials have been the primary source for discovering and studying quantum size effects, with examples of quantized excitation [4,5,6,7], Coulomb blockade [8], metal-insulator transition [9,10], and superparamagnetism [11].

The study of metallic materials is one of the most ancient scientific fields. Among all kinds of inorganic solids, metals deserve special attention because they represent more than two-thirds of the elements in the periodic table. Most metals crystallize in the same cubic close-packed (ccp) structure, a face-centered cubic (fcc) lattice that allows for easy characterization. Properties such as strength, toughness, thermal and electrical conductivities, ductility and high melting point make the metals useful for applications ranging from household items to rocket ships. Traditional applications are mainly based on the bulk metallic properties.

**Figure 1:**
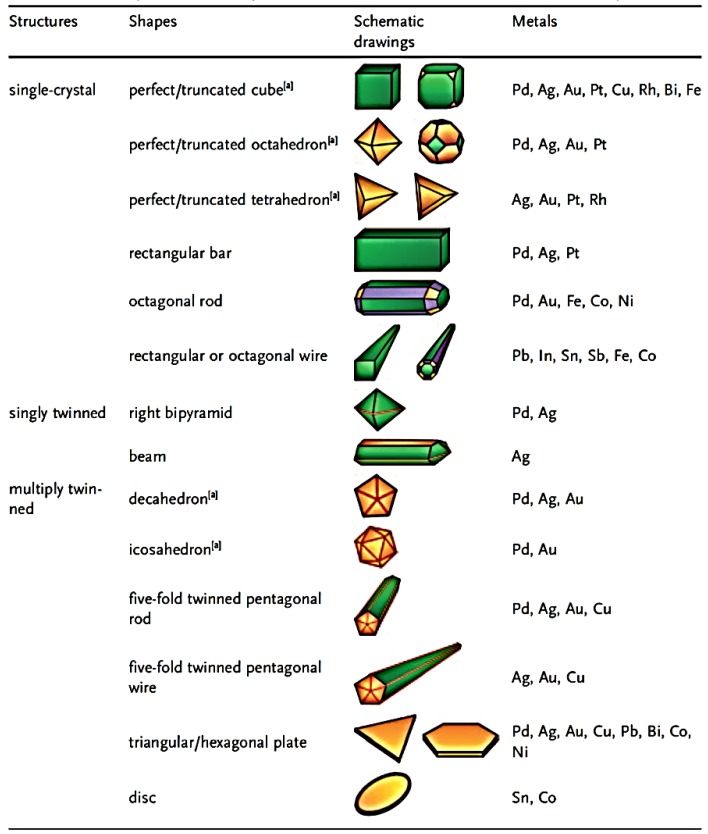
A summary of different shapes that have been achieved for various metal nanocrystals. Reproduced with permission from [2]. Copyright Wiley, 2008.

New applications exploit the novel properties of metal nanomaterials [12,13,14,15,16,17,18,19,20,21,22,23,24,25,26,27,28,29]. Nanomaterials exhibit fascinating size-, shape-, and crystal-form-dependent properties. Like the bulk metals, nanomaterials of metals are also going to bring profound changes in many spheres of science, technology, and industry. Though metal nanoparticles have a long history of preparation and applications, the field has undergone explosive growth in recent years. Metal nanoparticles with excess morphologies have already been prepared, such as polyhedrons, plates, prisms, rods, wires, nanoboxes, nanocages, dumbbells, nanoshuttles, stars, branched rods and wires, dendrites, nanorings, nanotubes, *etc*. Such metal nanostructures possess a range of advantageous properties, and many metals have found extensive use in applications that include catalysis [30], electronics [31], photography [32], and information storage [33,34], among others. New applications for metals in areas including photonics [14,16,17,18,19,35,36], sensing [37], imaging [38], and medicine [23,39] are also being developed. Significantly, most of these applications require the use of metals in a finely divided state, preferably in the form of nanocrystals with precisely controlled properties. The properties of a metal nano-crystal are determined by a set of physical parameters that may include its size, shape, composition, and structure. In principle, one can tailor the properties of a metal nanocrystal by controlling anyone of these parameters, but the flexibility and scope of change are highly sensitive to the specific parameter. For example, in the case of localized surface plasmon resonance (LSPR) and surface-enhanced Raman scattering (SERS), both computational and experimental studies have demonstrated that the shape and structure of Au or Ag nanocrystals play the most important role in determining the number, position, and intensity of LSPR modes, as well as the spectral region or polarization dependence for effective molecular detection via SERS [40]. In the case of catalysis, it is well established that the activity of a metal nanocrystal can be enhanced by reducing its size [41]. The selectivity, however, is most sensitive to the packing of atoms on the surface or the exposed facets of a nanocrystal [42]. For example, Pt can selectively catalyze different types of chemical reactions, with the {100} and {210} facets being most active for reactions involving H_2_ and CO, respectively [43]. The facets exposed by a nanocrystal clearly have a strong correlation with the shape. These and many other examples definitively illustrate the importance of shape control towards the efficient utilization of metal nanocrystals. The last decade has evidenced the successful synthesis of metal nanocrystals in a variety of shapes. Examples include: sphere; spheroid; cube; cuboctahedron; octahedron; tetrahedron; right bipyramid; decahedron; icosahedron; thin plate with a triangular, hexagonal, or circular profile; and rod or wire with a circular, square, rectangular, pentagonal, or octagonal cross-sections [2,44,45,46,47,48,49,50,51] (Figure 1). The emergence of many novel approaches induces synthesis and synthetic design, control of composition, size, morphology, and assembly structure and impressive advances in the characterization and manipulation techniques of metallic nanoparticles. A number of applications have been realized and multitudes of new applications have been envisaged. Among the many morphologies investigated, the anisotropic metal nanostructures have attracted a great deal of attention over the last decade due to their unique properties originating in their shape and their potential impact on new technologies.

By controlling the shape of the particle, the crystallographic planes that are exposed at the surfaces are tailored and, as a consequence, the atomic arrangement that makes the interface with the medium can be tuned. This has been shown to be critical for most chemical–physical properties, expanding the already broad range of applications of nanoparticles (NPs). One of the most appealing examples impacted by the shape-dependent properties of NPs is heterogeneous catalysis [52]. Several catalysts are composed by metal NPs dispersed on high surface area supports. In a heterogeneous catalytic reaction, the binding of the reagent molecules and the formation of the new bonds, which will form the product molecules, depend not only on the available orbitals of the reagents but also on the geometry of the atomic arrangement at the metal surface. Hence, controlling the exposed facets of an NP can be a major step toward a deeper understanding of the catalytic reaction and the design of better catalysts. Another key example in regards to the plasmonic-based behavior of noble metal nanostructures: metals such as gold and silver show a resonance in the ultraviolet-visible (UV-vis) region of the spectra known as surface plasmon [53]. This resonance arises from collective oscillations of the conduction electrons of the metal and strongly depends on the size, shape, local environment [14,16,17,18,19,24,54], and assembly of the NPs [55]. When a molecule is attached to the surface of a metal NP, its Raman scattering cross-section can be enhanced by several orders of magnitude, from 10^6^ up to 10^9^ if the incident light is tuned to the surface plasmon resonance of the particle [56]. This effect, known as SERS, results from an intense local amplification of the electric field near a metal surface when surface plasmon resonates in phase with the incident light. Because the electromagnetic near-field intensity is higher at sharp edges and tips, anisotropic NPs exhibit high Raman scattering enhancement factors that have been exploited for the detection of diluted molecules in analytical applications. For example, Mulvihill *et al.* [57] explored Langmuir–Blodgett monolayers of cubic, cuboctahedral, and octahedral silver NPs to detect trace amounts of arsenic in water. The best results were obtained with octahedral silver NPs, for which a detection level in the ppb range was achieved.

**Figure 2:**
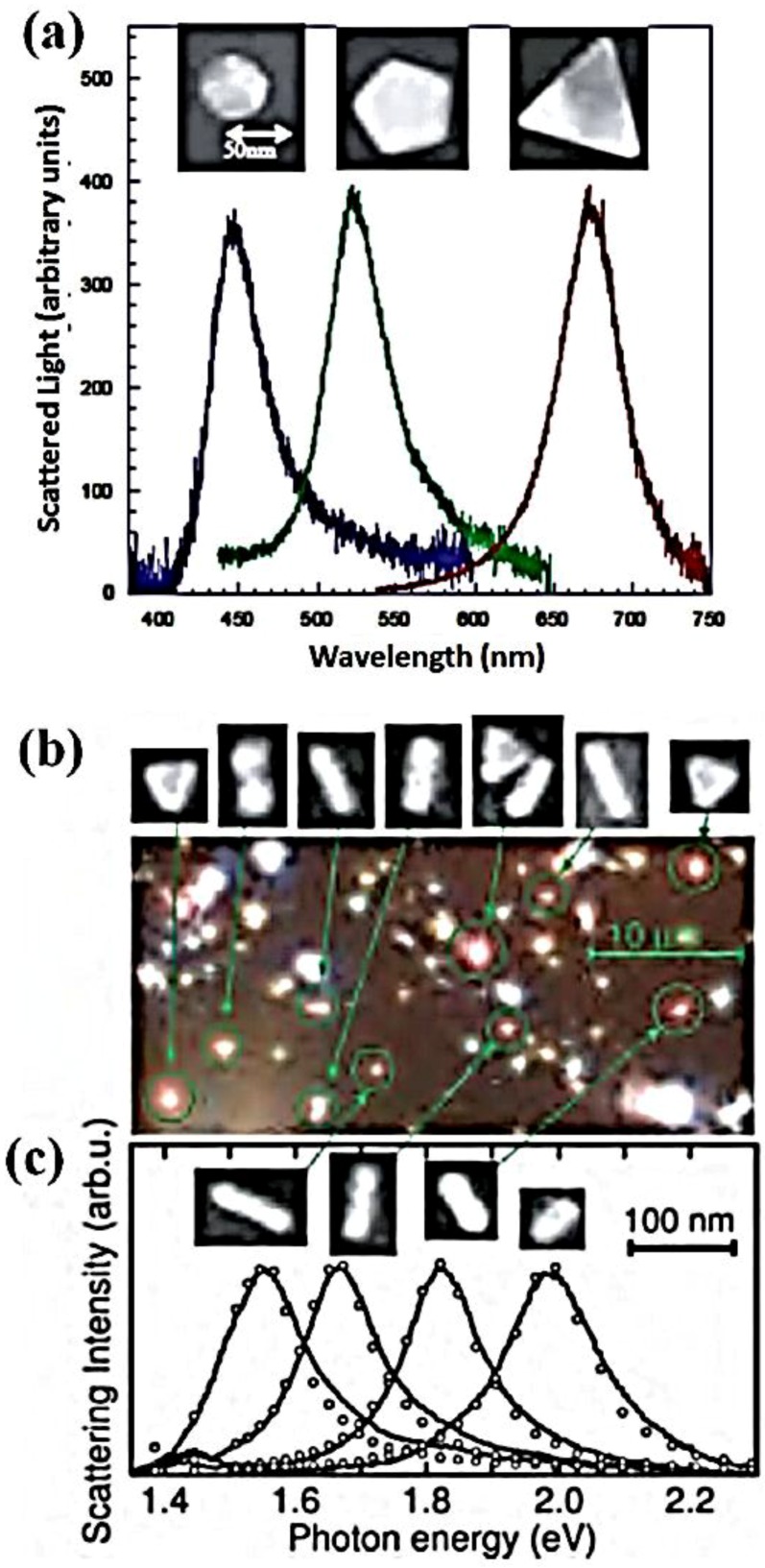
(**a**) Scattering spectra of single silver nanoparticles of different shapes obtained in dark-field configuration. Optical dark field images together with scanning electron microscope (SEM) images of individual gold nanoparticles (**b**) and corresponding scattering spectra (**c**) for an incident light polarization along the long particle axis. (**a**) Reproduced with permission from [59]. Copyright American Institute of Physics, 2002. (**b**,**c**) reproduced with permission from [60]. Copyright American Institute of Physics, 2003.

To understand the effects of the complex shape of the metallic nanoparticles on the plasmonic properties, the results of the optical characterizations related to non-regular geometries are shortly presented: for example, dark-field optical microscopy and near-field optical extinction microscopy enable the observation of plasmon resonances of a single particle. In dark-field optical microscopy, only the light scattered by the structure under study is collected in the detection path, while the directly transmitted light is blocked using a dark-field condenser. This enables the study of single particles dilutely dispersed on a substrate. Figure 2a shows an example of the dipolar plasmon line-shapes related to colloidal silver particles of different shapes. Conversely, in near-field optical spectroscopy, a thin (metalized or uncoated) fiber tip with an aperture to the order of 100 nm is brought into close proximity of the particle using an appropriate feedback scheme. The plasmon resonances can then be mapped out using either illumination through the tip and collection in the far-field, or evanescent illumination from the substrate side and light collection via the tip. Figure 2 also shows scattered light images (Figure 2b) and plasmon line-shapes (solid lines in Figure 2c) of a variety of gold nanoparticles [58,59,60].

**Figure 3:**
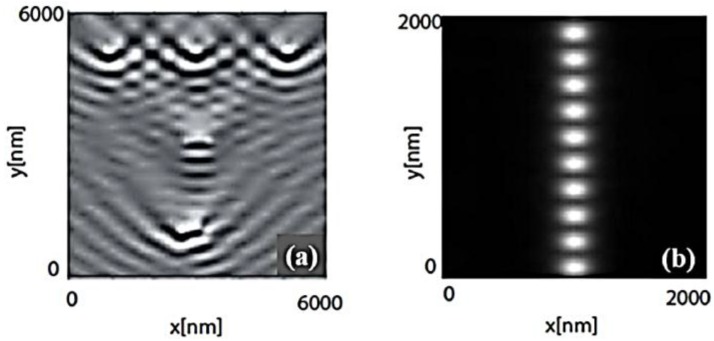
Simulated intensity distribution of the optical near-field above an ensemble of well-separated gold particles (**a**) and a chain of closely spaced gold nanoparticles (**b**). While for separated particles interference effects of the scattered fields are visible, in the particle chain, the fields are closely confined in gaps between adjacent particles. Plasmon resonances were excited using prism coupling with the direction of the in-plane moment component as outlined in the pictures. Reproduced with permission from [61]. Copyright Wiley, 2001.

Therefore, Figure 2 highlights that the localized plasmon resonance of a single metallic nanoparticle can be shifted in frequency from the Fröhlich frequency [58] via alterations in particle shape and size. Furthermore, in particle ensembles, additional shifts are expected to occur due to electromagnetic interactions between the localized modes. For small particles, these interactions are essentially of a dipolar nature, and the particle ensemble can, in a first approximation, be treated as an ensemble of interacting dipoles. Two regimes have to be distinguished, depending on the magnitude of the interparticle distance (d). For closely spaced particles, d << λ, near-field interactions with a distance dependence of d**^−^**^3^ dominate, and the particle array can be described as an array of point dipoles interacting via their near-field. In this case, strong field localization in nano-sized gaps between adjacent particles has been observed for regular one-dimensional particle chains [61]. The field localization is due to a suppression of scattering into the far-field via excitation of plasmon modes in particles along the chain axis, mediated by near-field coupling. Figure 3 illustrates this fact by showing the simulated distribution of the electric field above single gold nanoparticles (Figure 3a) and a particle chain (Figure 3b). From the images, it can clearly be seen that scattering is drastically suppressed for closely spaced particles, and that the fields are instead highly localized at interstitial sites. Interparticle junctions such as these therefore serve as hot-spots for field enhancement, which will be discussed later. Therefore, the interparticle coupling will lead to shifts in the spectral position of the plasmon resonance compared to the case of an isolated particle.

**Figure 4:**
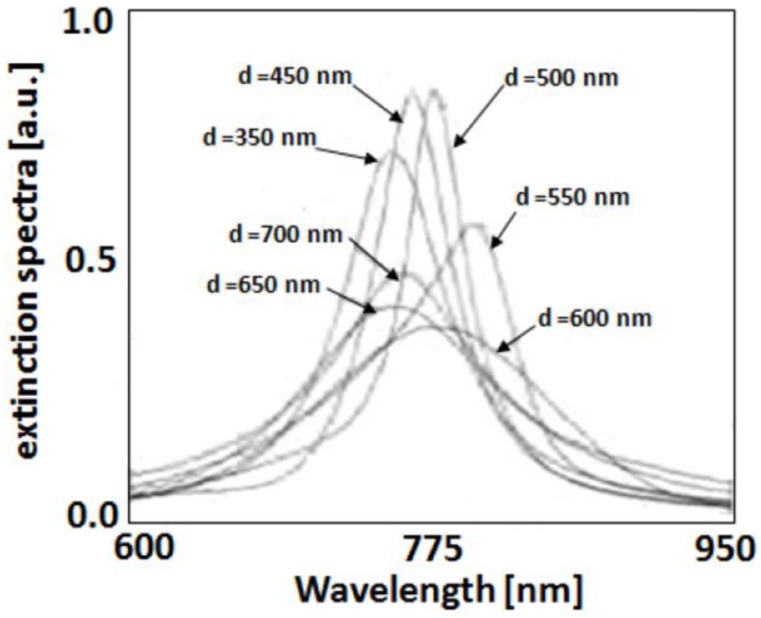
Extinction spectra for square two-dimensional gratings of gold nanoparticles (height 14 nm, diameter 150 nm) with grating constant d situated on a glass substrate. Reproduced with permission from [63]. Copyright American Physical Society, 2000.

Maier *et al.* [62], using one-dimensional arrays of 50 nm gold particles with varying interparticle distance, demonstrated experimentally the shifts in resonance energy using far-field extinction spectroscopy. Due to the strong scaling of the interaction strength with d**^−^**^3^, particle separations in excess of 150 nm are sufficient to recover the behavior of essentially isolated particles. For larger particle separations, d >> λ, far-field dipolar coupling with a distance dependence of d**^−^**^1^ dominates. For example, two-dimensional gratings of gold nanoparticles with various lattice constants [63] (Figure 4) shows that far-field coupling has pronounced influences on the plasmon line-shape, both in terms of resonance frequency as well as spectral width. We note that interactions between metal nanoparticles can be further enhanced by providing additional coupling pathways as, for example, in the form of propagating Surface Plasmon Polariton (SPPs) for particle arrays fabricated on a conductive substrate.

These examples show how the optical response of isolated metal nanostructures or metal nanostructure assemblies are strongly dependent on the nanostructure morphology and topology. The rationale design of the morphology and topology of the nanostructures can give the possibility to confer a specific functionality to the nanostructures in relation to its interaction with the electromagnetic radiation. For example, a single nanostructure of complex morphology can be characterized by several hot-spots with respect to the interaction with the electromagnetic radiation. The same number of hot-spots are, instead, obtainable by a large number of spherical nanoparticles assembled in a specific array.

Starting from these general ideas, in the following sections we report synthesis methods and property characterization for some peculiar metal nanostructures with complex shapes and composition in view of advanced applications in the field of electronic, opto-electronic, sensing, bio-sensing and magnetic devices.

## 2. Gold Nanorings and Ring Dimers

**Figure 5:**
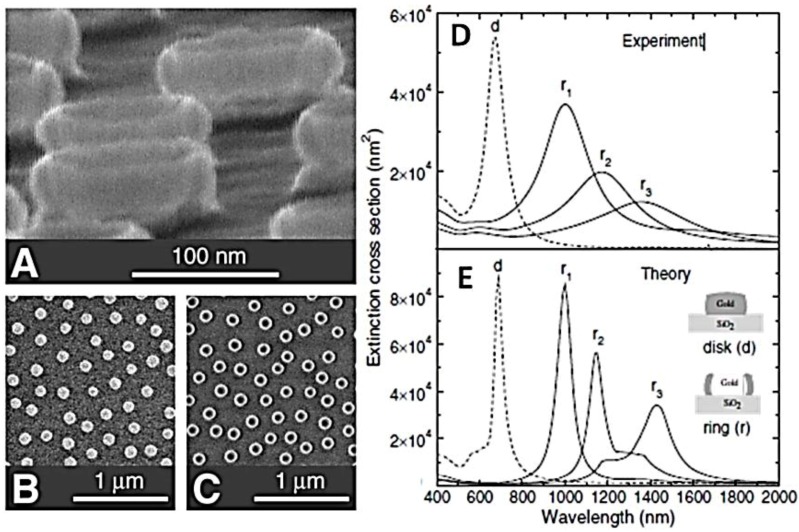
SEM images of gold nanorings and nanodisks prepared by colloidal lithography. (**a**) 80° tilt image of a ring structure. The walls of the rings are thin enough for the 30 keV electrons to pass through; (**b**,**c**) Top views of disks and rings taken at an acceleration voltage of 1.5 keV. The heights of the disks and rings in the figure are **≈**20 and **≈**40 nm, respectively, whereas the radius is **≈**60 nm in both cases. The thickness of the ring walls was estimated at 14 ± 2 nm from sideview SEM images similar to (**a**); (**d**) Experimental extinction spectra of disks (*d*, dashed line) and rings (*r*, solid lines). The estimated wall thickness d of the rings is *d* = 14 ± 2 nm (*r*_1_), d *=* 10 ± 2 nm (*r*_2_), and d = 9 ± 2 nm (*r*_3_), respectively. (**d**) Calculated extinction spectra for the same particles as in (**a**). The rings exhibit near-infrared features at larger wavelengths for thinner walls. The disks show a dipolar excitation at around 700 nm. Reproduced with permission from [69]. Copyright American Physical Society, 2003.

The field enhancement around nanoscale metallic particles offers an efficient tool to manipulate light–matter interactions at defined optical frequencies [64,65]. For spectroscopic applications, the field confinement around these structures reduces the mismatch between the wavelength of the incident light and size of the molecules under study. In the SERS, this can boost the Raman cross-section of molecules by many orders of magnitude. SERS offers a very sensitive tool for biological and chemical sensing [66], enabling vibrational spectra to be obtained even from single molecules [67]. The signal enhancement in SERS mostly comes from the excitation of localized surface plasmon resonance (LSPRs) and the “lightning rod” effect [68]. The latter is particularly pronounced for sharp tips and nanoparticle dimers with small inter-gap size. The optimum substrate for SERS should have the highest average field enhancement possible, be highly tunable, and offer a broad resonance bandwidth, although satisfying all these criteria is very challenging. Promising candidates as highly tunable nanostructures fulfilling these requirements are metal nanostructure characterized by a ring geometry [13,69]. The optical properties of gold nanorings fabricated by colloidal lithography were reported by Aizpurua *et al.* [69]. They showed that the LSPR frequency strongly depends on the ratio of the thickness to ring outer radius. This tunability, which is similar to that of nanoshells [70], allows the control of the plasmon resonances from visible to near-IR wavelengths via reducing the ring thickness. The ring-like nanoparticles (Figure 5a,c) exhibit tunable plasmon resonances in the near infrared that are not observed for solid particles of similar size (nanodisks in Figure 5b). The volume confined by the nanoring is empty and therefore accessible to various sensing and spectroscopy applications at the nanometer scale. The experimentally observed optical response of these structures is well described by numerical simulations (Figure 5d) and the main features can be qualitatively understood from simple models of the charge oscillation patterns. The predictive character of these calculations allows one to tailor the shape of a particle in order to achieve excitation spectra on demand with controlled field enhancement. The reduced amount of the metal in the ring geometry compared to disks of the same radius results in smaller absorption loss. Fabricating metallic rings with high-resolution methods such as electron beam lithography enables a great control of the periodic arrangement of the nanoparticles. Moreover, the ring geometry has an advantage over the nanoshell pattern in that molecules can attach to the inner wall as well as the outer wall, which is beneficial as substantial field enhancement occurs there. In addition to the conventional SERS, studying the field confinement in metallic rings could also have applications in optical corrals [71] and lasing spacers [72].

Banaee *et al.* [64] compared the SERS spectra related to single nanoring and nanoring dimmers with that of an array of nanodisk dimers. For SERS measurement, self-assembled monolayers of benzenethiol are formed on the gold nanostructures. To compare SERS enhancements of ring and ring dimer structures with the disk dimmers, similar steps are taken for the measurement of the latter. The experimental SERS enhancement factor (EFs) for 1074 cm**^−^**^1^ line of benzenethiol for all patterns are measured and that of the ring and dimer rings results in 3.9 × 10^6^ and 4.2 × 10^6^, respectively, and they are roughly seven times higher than the dimer disks.

Even if the nanoring architecture presents several strengths with respect the nanoshell one, this last resulted of primary importance when its plasmonic properties were combined with those of the nanorod architecture in a single structure, named “nanorice”, as discussed in the following section.

## 3. Gold Nanorices

**Figure 6:**
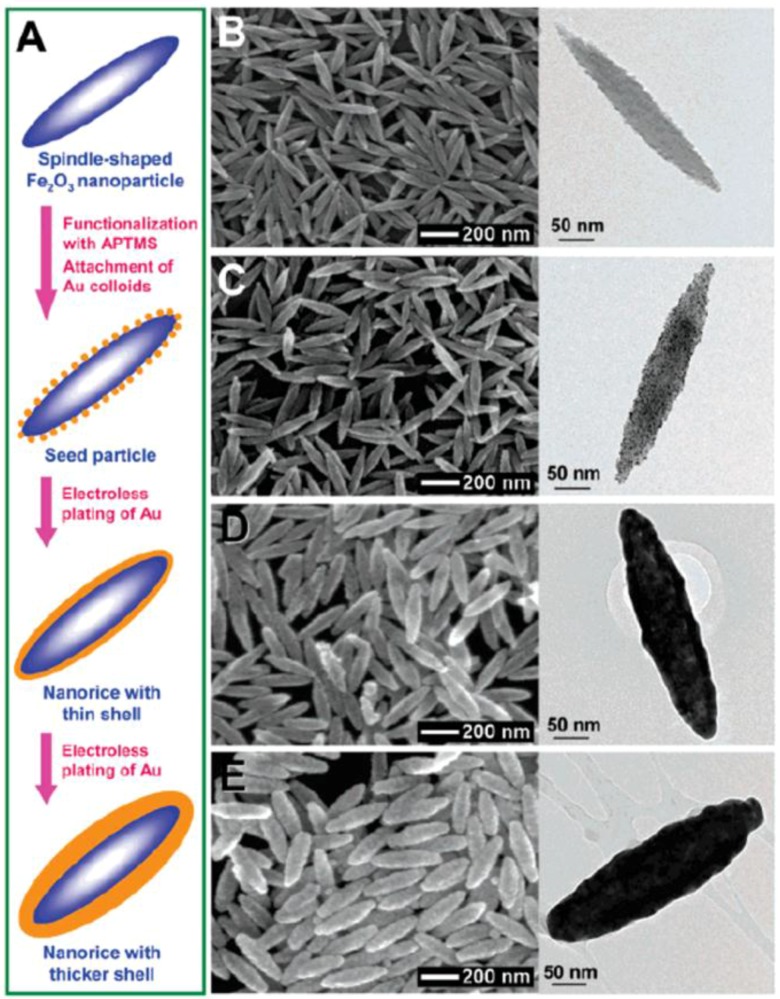
(**A**) Schematics of the fabrication of hematite-Au core-shell nanorice particles. SEM (**left**) and transmission electron microscopy (TEM) (**right**) images of (**B**) hematite core (longitudinal diameter of 340 ± 20 nm, and transverse diameter of 54 ± 4 nm; (**C**) seed particles; (**D**) nanorice particles with thin shells (13.1 ± 1.1 nm); and (**E**) nanorice particles with thick shells (27.5 ± 1.7 nm). Reproduced with permission from [73]. Copyright American Chemical Society, 2006.

This new hybrid nanoparticle geometry combines the plasmonic properties of both nanorods and nanoshells in a single structure. This dielectric core-metallic shell prolate spheroid nanoparticle bears a remarkable resemblance to a grain of rice, inspiring the name “nanorice”. Wang *et al.* [73] report nanorice (see Figure 6) composed of a spindle-shaped hematite core coated with a continuous nanometer-thick Au shell. To apply the plasmon hybridization picture to analyze the resonances of this layered nanoparticle geometry, the authors analyze the plasmon resonances of a solid metallic nanorod and a nanocavity of arbitrary ellipticity as the parent plasmons which, when hybridized, give rise to the plasmon resonances of nanorice. The fabrication of nanorice involves seeded metallization of spindle-shaped hematite nanoparticle cores (Figure 6A. Small Au nanoparticles (~2 nm in diameter) are immobilized onto the surface of (3-aminopropyl) trimethoxysilane (APTMS) functionalized cores at a nominal coverage of ~30%. The immobilized Au colloids act as nucleation sites for electroless Au plating onto the surface of core particles, leading to the gradual formation of a continuous and complete Au shell layer. This is essentially the same metallization procedure used in silica−Au nanoshell synthesis [74] and shows that this approach is readily adaptable to produce uniform metallization layers on the surfaces of other oxide nanoparticles. Further metal deposition onto the nanostructure increases the thickness of the metal layer. Nanorice can be dispersed homogeneously in solvents such as water and ethanol to form colloidal solutions or be dispersed and immobilized on polyvinylpyridine (PVP)-coated substrates as individual, randomly oriented nanoparticles.

**Figure 7:**
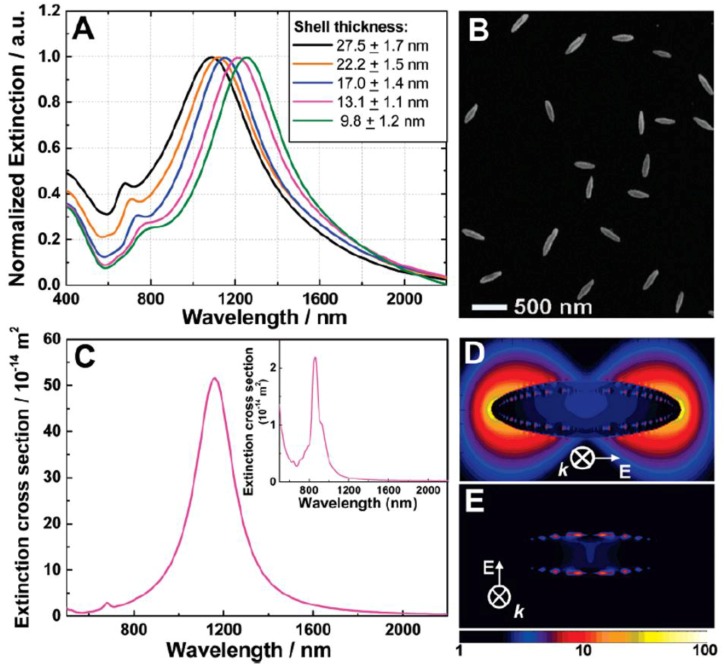
(**A**) Extinction spectra of hematite-Au core-shell nanorice with different shell thicknesses. Two plasmon peaks are observed for each sample. The plasmons at longer and shorter wavelengths are the longitudinal and transverse plasmons, respectively. The samples measured are monolayers of isolated nanoshells immobilized on polyvinylpyridine (PVP)-glass slides; (**B**) A SEM image of a monolayer of nanorice particles (shell thickness of 13.1 (1.1 nm) on a PVP-glass slide; (**C**) Calculated far-field extinction spectra of the nanorice with incident polarization along the longitudinal and (inset) transverse axis of a nanorice particle using finite difference time domain (FDTD). The nanorice particle employed for the FDTD simulations is composed of a hematite core with longitudinal diameter of 340 nm and transverse diameter of 54 nm surrounded by a 13-nm-thick Au shell. Near-field profile of the nanorice under resonance excitations: (**D**) incident polarization along the longitudinal axis, λ_ex_ = 1160 nm; (**E**) incident polarization along the transverse axis, λ_ex_ = 860 nm. Reproduced with permission from [73]. Copyright American Chemical Society, 2006.

Extinction spectra of nanorice with varying thicknesses of the shell layer are shown in Figure 7A. Representative SEM image of the nanorice sample used to obtain these optical measurements is shown in Figure 7B. The strong plasmon resonance feature observed in the spectra in Figure 7A arises because of the longitudinal plasmon of this layered structure and exhibits a highly sensitive structural dependence of its optical resonance, which blueshifts as the metal layer thickness is increased. A finite difference time domain (FDTD) analysis [75] of the far field extinction spectrum of this nanostructure reveals that the transverse plasmon mode (Figure 7C inset) has a much weaker extinction cross-section than the longitudinal mode of this nanostructure (Figure 7C. The weaker, higher energy resonance observed in the spectrum of nanorice is attributable to the far weaker transverse plasmon mode, with a small additional contribution from a higher order longitudinal plasmon mode. The local fields associated with the longitudinal and transverse nanorice plasmons are shown in Figure 7D,E. The asperities of this structure support very strong local field intensity enhancements (>7000 for this specific nanorice geometry) at wavelengths corresponding to the longitudinal plasmon resonance of the nanostructure. This intensity enhancement is several times larger than those reported for nanofabricated bowtie junctions [76] and what has been predicted and measured in scanning probe junctions [77,78]. Such strong, tunable local fields make this geometry highly attractive for use in designing substrates for surface-enhanced spectroscopy-based sensing. These large plasmon-resonant local field enhancements are similar in magnitude to the localized plasmon resonant “hot-spots” occurring in junctions between metallic nanoparticles when a dimer plasmon resonance is excited [79,80]. The nanorice local fields should give rise to intense SERS enhancements with the added advantage that the hot-spots are completely open to the surrounding medium in this geometry. From this point of view, each nanorice particle can potentially serve as a standalone, optically addressable nanoscale substrate for surface-enhanced spectroscopies. Moreover, because the enhanced near-field intensities can extend several tens of nanometers from the surface of the nanorice, these particles may exhibit unique advantages in the spectroscopic sensing and characterization of large biomolecules, such as proteins and DNA, biological samples, or materials placed directly adjacent to the nanoparticle.

The nanorice architecture is the simplest example of how hot-spots can originate in a single nanostructure simply deforming the spherical geometry. The next example, the nanoporous gold architecture, shows as the topological complexity on the nanoscale allows the establishment of a high number of hot-spots in an extended nanostructured system giving rise, at the same time, to a system with an enormous exposed surface area which is beneficial for ultrasensitive devices.

## 4. Nanoporous Gold Films

Porous materials have recently been attracting considerable attention because of a wide range of applications in catalysis, sensing, micro/nanoelectromechanical systems, and biotechnology [81]. One example is nanoporous gold formed by chemically or electrochemically dealloying of silver−gold alloys [82,83,84,85], which possesses bi-continuous porosity and excellent electrical and thermal conductivities. Nanoporous gold offers an extensive surface where the analytes can connect, thereby increasing sensitivity and efficiency [86]. Nanoporous gold (NPG) may be formed by a spontaneous pattern-forming instability during the chemical etching of silver from gold−silver alloys [87]. The leaching of the less noble metal gives rise to a bi-continuous sponge-like structure of nanopores and gold ligaments [88] whose geometric features depend on the alloy composition and on the experimental conditions of the dealloying process [89]. The nanoporous structure affects also the optical properties: the plasma frequency ω_p_ exhibits red-shift due to the lower density in comparison with bulk gold and the material shows metallic behavior for wavelengths above the near-IR range [90]. Nanoporous gold with excellent thermal stability and chemical inactivity has recently been exploited as an attractive substrate for SERS applications because of its large surface area and bi-continuous porous structure in three dimensions [91]. These SERS-active substrates combine the self-organized and highly interacting nanoscale morphology of NPG with the advantages of reproducibly nanopatterned periodic structures. Nevertheless, conventional nanoporous gold with a pore size of about tens of nanometers made by room temperature dealloying does not show a promising SERS enhancement although nanostructured gold, such as gold nanoparticles, are known to be SERS active for a number of organic and biological molecules [92]. Thus, optimizing the microstructure is crucial to the improvement of the SERS enhancements and thus the SERS application of nanoporous gold, which has been attempted by a few of research groups [93,94,95]. More recently, Kucheyev *et al.* [94] reported that the optimized SERS enhancement can be achieved in the annealed nanoporous gold with a coarsened pore size of ~250 nm. However, Qian [96] found that the strongest SERS enhancement takes place from the nanoporous gold with an ultrafine nanopore size of ~5–10 nm. The correlation between the structural parameters of the NPG films and the improvement of the optical properties drives the search to optimize the synthesis processes in order to obtain a greater control over the size of the nanopore structures. Subsequently, we report synthesis methods used to obtain porous materials with a greatly enhanced surface-to-volume ratio which is particularly suitable for the realization of plasmonic supports for sensing purposes.

**Figure 8:**
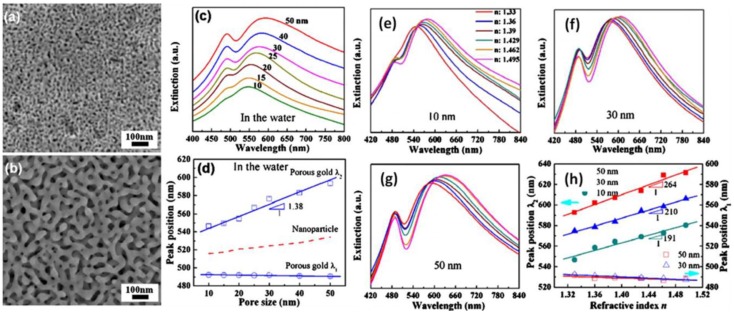
SEM micrographs of NPG synthesized by means of corrosion for 5 min (**a**) and 24 h (**b**); (**c**) ultraviolet (UV)-vis extinction spectra and (**d**) the resonant peak position of λ_1_ and λ_2_ of NPG with the pore sizes of 10–50 nm in water. For comparison, the dashed line represents the size dependence resonant band of gold nanoparticles. UV-vis extinction spectra of porous gold films with (**e**) 10 nm; (**f**) 30 nm; and (**g**) 50 nm are recorded by immersing them into various dielectric environments. Refractive index of these solutions increase from left to right: water (*n* = 1.33), ethanol (*n* = 1.36), 3:1 ethanol/toluene (*n* = 1.39), 1:1 ethanol/toluene (*n* = 1.429), 1:3 ethanol/toluene (*n* = 1.462), and toluene (*n* = 1.495). (d) Dependence of resonance (λ_1_), empty symbols and LSPR (λ_2_, solid symbols) peaks of NPG films on refractive index. Reproduced with permission from [97]. Copyright American Institute of Physics, 2011.

Lang *et al.* [97], report the plasmonic properties of free-standing nanoporous gold films with an intricate bicontinuous nanostructure. Ag_65_Au_35_ (atomic ratio) leaves with a size of ~20 mm × 20 mm × 100 nm were dealloyed in a 70% (mass ratio) HNO_3_ solution at room temperature. Nanopore sizes can be tailored from 10 to 50 nm by controlling the corrosion time in the range of 5 min to 24 h (Figure 8a,b). Intermediate porous structures are quenched by distilled water, and residual acid within nanopore channels is removed by water rinsing. UV-vis extinction spectra of the NPG films with pore sizes of 10, 15, 20, 25, 30, 40, and 50 nm soaked in the water are shown in Figure 8c. Regardless of nanopore sizes, two characteristic resonant bands can be detected in all the NPG samples. One at ~490 nm, resulting from the resonant absorption of gold films, is independent of nanopore sizes and dielectric surroundings. The other at ~550–650 nm, arising from the excitation of localized surface plasmon resonance, shows obvious band shift with the nanopore sizes and dielectric indices of surrounding media, suggesting that NPG is a promising candidate as plasmonic sensors for organic and biologic molecule detection. The spectral features of NPG are apparently different from those of other nanostructured golds. For instance, gold nanoparticles are characterized by an isolate surface plasmon resonance (SPR) band and gold nanorods exhibit one transverse plasmon band at ~520 nm [98,99]. The low wavelength peak of NPG at 490 nm originates from the resonant absorption of gold films [100]. The resonant location mainly relies on film thickness rather than nanopore and ligament sizes. Thus, it is nearly independent of nanopore sizes. In contrast, the high wavelength band, arising from LSPR, represents the significant redshift with the increase in pore sizes, which expectedly results from the effective electron oscillation lengths that are determined by the gold ligament sizes. Due to these intense SPR peaks, the authors tested the produced NPG films as plasmonic sensors. Figure 8a–c show the extinction spectra of the NPG films immersed into a series of organic dielectric media. The SPR peak at 490 nm (λ_1_) does not show a detectable shift when the refractive indices of the media (n) increase from 1.33 to 1.495. In contrast, the SPR band at ~540 nm (λ_2_) represents evident redshift with the refractive indices (see Figure 8e–g). The shift magnitude of λ_2_ has a linear relationship with the refractive index as shown in Figure 8h. For the λ_2_ SPR band, the NPG films with larger pore sizes show more significant redshift with the refractive index. Thus, the refractive index sensitivity of NPG is comparable to those of other nanostructured golds, such as gold nanorods [99], hollow sphere and cylindrical holes.

**Figure 9:**
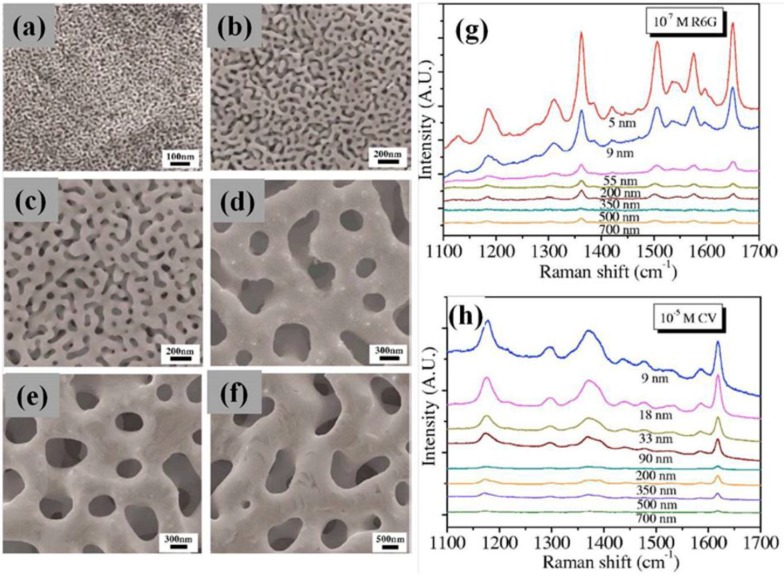
Representative SEM micrographs of nanoporous gold with various nanopore sizes. (**a**) Nanoporous gold film after 5 min dealloying at room temperature; (**b**) dealloyed at room temperature for 48 h; (**c**) thoroughly rinsed nanoporous gold annealed at 200 °C for 2 h; (**d**) 400 °C for 2 h; (**e**) 500 °C for 2 h; and (**f**) 600 °C for 2 h. SERS spectra of nanoporous gold with different pore sizes for (**g**) 10^−7^ mol/L R6G aqueous solution and (**h**) 10^−5^ mol/L crystal violet (CV) methanol solution. Reproduced with permission from [96]. Copyright American Institute of Physics, 2007.

Qian *et al.* [96] report, instead, the results of the characterization of two classes of Ag_35_Au_65_ (atomic %, 50/50 by weight) films with the thicknesses of ~100 nm and ~60 μm, respectively, used to synthesize nanoporous gold by selective chemical and electrochemical dealloying in 70% HNO_3_ aqueous solution at the temperatures of −20, 0, and 25 °C, respectively. By controlling the dealloying time and temperatures, the authors prove that the nanopore sizes can be tailored from ~5 to ~33 nm for 100 nm thick films (see SEM images in Figure 9a,b). The 60 μm thick nanoporous gold with a pore size of ~55 nm, produced by room-temperature dealloying for 48 h, is further annealed at 200, 300, 400, 500, and 600 °C for 2 h in air, which results in the coarsening of nanoporous gold with pore sizes ranging from 90 to 700 nm. The nanoporous gold specimens with various nanopore sizes were subjected to SERS experiments with rhodamine 6G (R6G) and crystal violet (CV) 10B as test molecules (see SEM images in Figure 9c–f). SERS enhancements of nanoporous gold for both R6G and CV molecules prove that ultrafine nanopores possess the strongest SERS enhancement. Although high enhancements can be obtained in the annealed samples with a large pore size of 350 nm, similar to the observations of Kucheyev *et al.* [94], this anomalous enhancement originates from the ultrafine pimple-type irregularities on the rough gold surfaces. Thus, the observations of the SERS enhancements of nanoporous gold confirm that the high SERS enhancements result from smaller microstructure features, either smaller pore sizes or the rough gold ligament surfaces, which are believed to promote electromagnetic field enhancements and provide more active sites for molecule adsorption [101,102]. Because the formation and coarsening of nanoporous gold are controlled by surface diffusion, the smaller pore sizes formed by a short dealloying time at low temperatures generally contain more or less residual silver. It is known that the SERS effect of silver exceeds that of gold [103]. Thus, the stronger Raman scattering from the ultrafine nanoporous gold may also include the extra contribution from the residual silver. However, the fact that the annealed nanoporous gold (without detectable residual silver) containing a high density of surface irregularities exhibits the analogous enhancement as the ultrafine nanoporous gold indicates that the ultrafine structures, both small pore sizes and fine surface pimple-type irregularities, play the major role in the strong SERS enhancement of the nanoporous gold.

Chen *et al.* [95] studied the plasmonic properties of a 20-nm-thick nanoporous Au film by far-field and near-field optical techniques. The film is prepared sequentially by deposition of gold and copper, thermal annealing, and chemical etching, and has randomly distributed nanopores with sizes ranging between 20 and 350 nm. The nanoporous film has a different transmittance and a lower reflectance when compared with those of a 20-nm-thick plain Au film in the wavelength range between 400 and 1000 nm. As a result, the absorbance of the nanoporous film is much higher and can be attributed to the conversion of incident light into SPPs. In the dark-field scattering spectrum, a broad peak appears at around 630 nm and confirms the presence of HPR (Hole Plasmon resonances) [104] of the nanoporous film. With the aid of transmission mode NSOM (Near Scanning Optical Microscopy), local field distribution on the film is observed and reveals the generation of SPPs. Furthermore, two types of local field enhancement with spatial extents of around 200 nm and 1 nm are observed. The two types of enhancement correlate with strong and weak SPP localizations, respectively.

The peculiar optical properties of this complex-morphology structure are highlighted by the simulation reported by Lang, using a discrete dipole approximation (DDA) [105]. A simplified nanostructure representing the key structural features of NPG is introduced to qualitatively simulate the optical properties of NPG with the bulk dielectric function of Au [106]. Under the plane wave with a wavelength of 514 nm propagating along the direction normal to the top surface of the nanostructure, the total near-field E^2^/E_0_^2^ distributions are calculated with pore size *d* = 10 nm and Au ligaments size *D* = 30 nm, and also with *d* = *D* = 10 nm, and *d* = *D* = 20 nm, respectively, exhibiting that E^2^/E_0_^2^ increases with the decrease in the *d*/*D* ratio. This is qualitatively in accordance with experimental measurements of the SERS enhancements reported in many works. The electromagnetic field enhancements from near-field coupling dramatically increase as the ratio of *d*/*D* decreases compared to the NPG with the same pore size but large *d*/*D* ratios. Therefore, for SERS effect of NPG films, in addition to the LSPR, the weakening of plasmon damping with increasing ligament sizes obviously enhances the near-field coupling between neighboring ligaments, which gives rise to the observed SERS improvements with the decrease in the *d*/*D* ratios.

**Figure 10:**
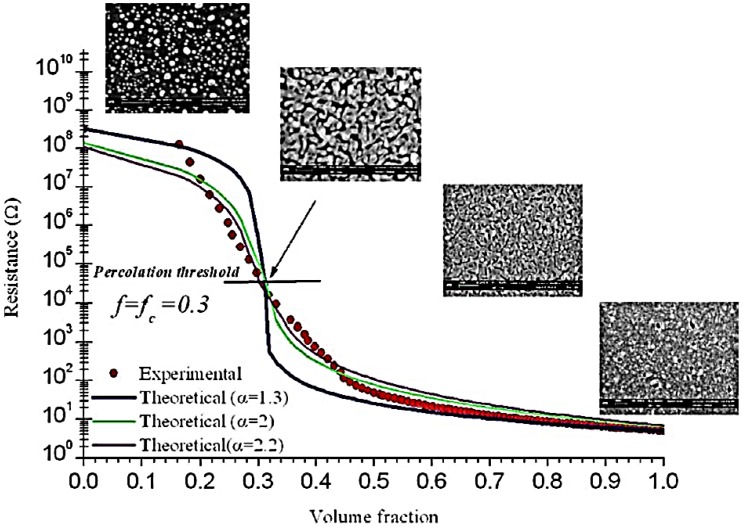
Resistance (dots are the experimental data) of a growing gold film on glass as a function of volume (or area) fraction f of gold. Images included show characteristic morphology at each stage of gold coverage. Reproduced with permission from [107]. Copyright American Physical Society, 2008.

For the sake of completeness, we mention that in ideal conditions, nanoporous gold films can be produced without compromising its electrical performance. In fact, also the electrical transport properties of nanoporous gold films are widely studied [107,108,109,110]. Smith *et al.* [107] analyzed samples of nanoporous gold of various morphologies by a combination of electrical and optical data. Concerning the electrical response, the authors reported the resistance of a growing gold film on glass as a function of volume (or area) fraction *f* of gold (Figure 10), finding a percolation-type behavior. This resistance data is consistent with a percolation threshold of *f*_c_ = 0.31 ± 0.03.

We highlight that Chen *et al.* [111] exploited the electrical properties of nanoporous gold films to fabricate a non-enzyme electrochemical glucose sensor: The free-standing NPG films showed a robust and sensitive current response to glucose. The authors also investigated effects of pore sizes and detecting potentials. Chloride ions on glucose oxidation were systematically investigated finding that NPG with a smaller pore size possesses higher sensitivity.

**Figure 11:**
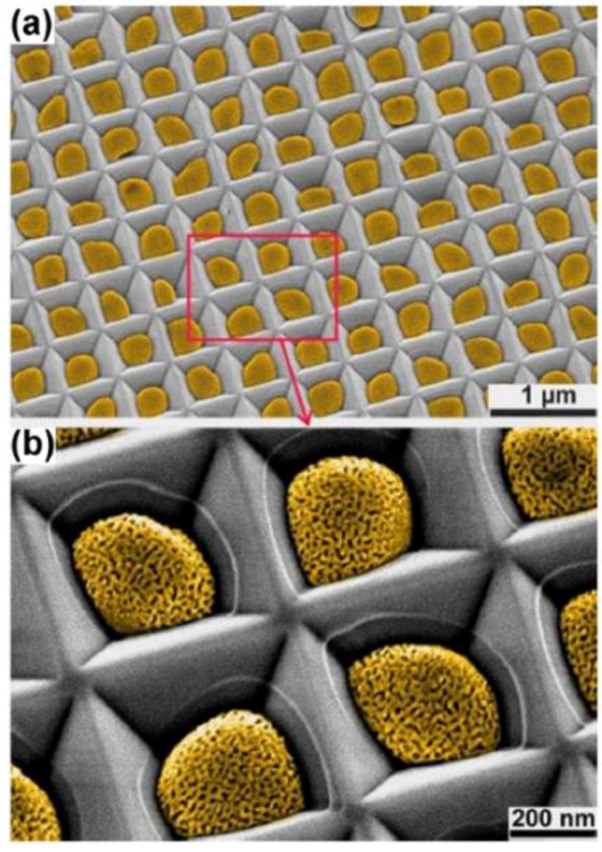
SEM images (false color) at 25° tilt of the perfectly ordered array of the nanoporous gold nanoparticles formed from the 15 nm Au/30 nm Ag bilayers. Reproduced with permission from [112]. Copyright Beilstein-Institut, 2012.

**Figure 12:**
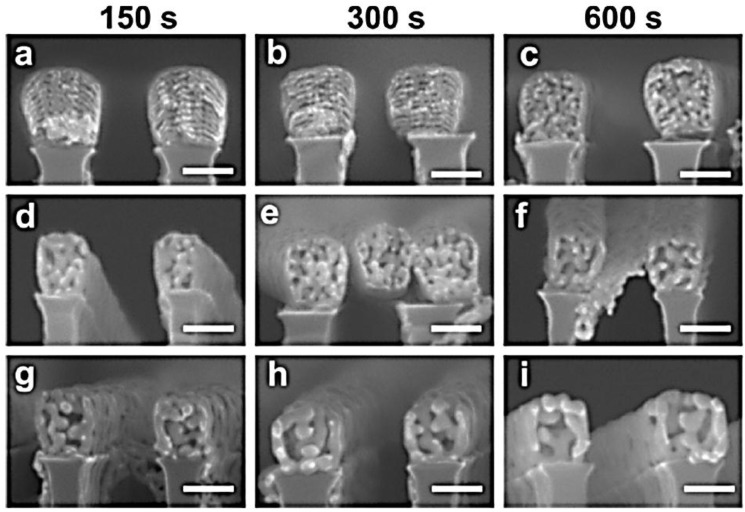
Cross-sectional SEM micrographs showing the morphological evolution of Au–Cu nanowires with an initial Au content of 11 atomic % and a diameter of 200 nm as a function of the dealloying time and potential. Different dealloying potentials were explored: (**a**–**c**) 0.2; (**d**–**f**) 0.3; and (**g**–**i**) 0.4 V. Scale bar: 100 nm. Reproduced with permission from [113]. Copyright American Chemical Society, 2016.

Recent research has likewise been devoted to the fabrication of porous Au nanostructures such as porous Au nanoparticles [112] or nanowires [113]. For example, Wang *et al.* [112] developed a combination of a “top-down” approach (substrate-conformal imprint lithography) and two “bottom-up” approaches (dewetting and dealloying) for the fabrication of perfectly ordered 2D arrays of nanoporous gold nanoparticles (Figure 11). The dewetting of Au/Ag bilayers on the periodically prepatterned substrates leads to the interdiffusion of Au and Ag and the formation of an array of Au–Ag alloy nanoparticles. The array of alloy nanoparticles is transformed into an array of nanoporous gold nanoparticles by a following dealloying step in HNO_3_. This technique allows for the control of particle size, particle spacing, and ligament size (or pore size) by varying the period of the structure, total metal layer thickness, and the thickness ratio of the as-deposited bilayers. Chauvin *et al.* [113] developed a two-step approach allowing creation of highly ordered porous gold nanowire arrays with a length up to a few centimeters. This two-step approach consists of the growth of gold/copper alloy nanowires by magnetron cosputtering on a nanograted silicon substrate, serving as a physical template, followed by a selective dissolution of copper by an electrochemical anodic process in diluted sulfuric acid. The authors demonstrated that the pore size of the nanowires can be tailored between 6 and 21 nm by tuning the dealloying voltage between 0.2 and 0.4 V and the dealloying time within the range of 150–600 s. In addition, they showed that the initial gold content (11 to 26 atomic %) and the diameter of the gold/copper alloy nanowires (135 to 250 nm) are two important parameters that must carefully be selected to precisely control the porosity of the material (Figure 12).

The nanorice and nanoporous architectures highlighted the key role played by the “hot-spots” in determining very high enhancement factors in SERS performances. Now, the question which arises concerns how to maximize the number of hot-spots in a single nanostructure. A nanorice architecture generates a low number of hot-spots. Nanoporous gold can create a very high number of hot-spots which, however, derive in an extended configuration by the interaction of several gold ligaments. Therefore, we are now looking for a single defined nanostructure which, thanks to the complexity of its geometry, can give rise in a confined spatial region to a very high number of hot-spots. As described in the next section, this is due to nanodendritic geometry.

## 5. Metallic Nanodendrites

**Figure 13:**
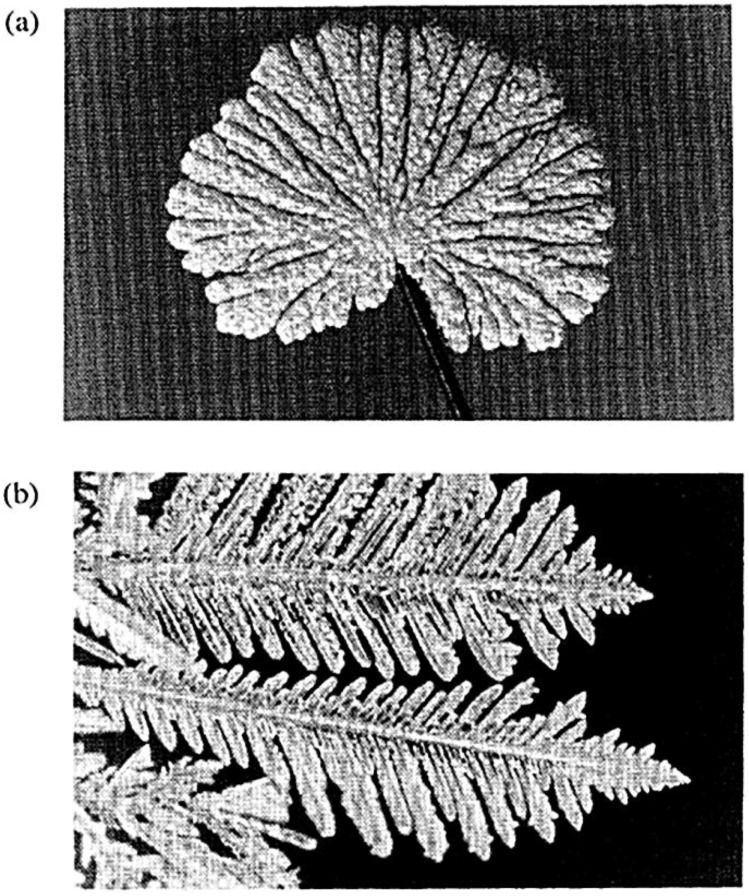
Dendritic structures obtained by electrochemical deposition of copper. (**a**) From 0.5 M CuSO_4_/0.5 M H_2_SO_4_ at 350 mV (frame width = 2.5 mm); and (**b**) from 0.5 M CuCl_2_ /0.5 M H_2_SO_4_ at 500 mV (frame width = 0.6 mm). Reproduced with permission from [114]. Copyright American Physical Society, 1995.

The “lightning-rod” effect [68] is particularly pronounced for metal nanostructures with sharp tips and with small inter-gap size. In this sense, one of the most promising candidates for a highly tunable SERS system is the dendritic architecture. Metallic nanodendrites, defined as large fractal aggregates with hyperbranched architectures (see Figure 13) have attracted much attention due to their importance in understanding the fractal growth phenomena (to be distinguished from non-fractal structures such as compact or periodic geometries), and their potential applications in functional devices, plasmonics, biosensing and catalysis.

Fractal nanostructures can be formed by metals (Au [115,116], Pt [117,118], Pd [119,120], Rh [121,122], Ni [123], Ru [124,125]), metal oxides [126,127,128,129], and semiconductors [130,131,132,133,134,135,136]. Because of their higher structural complexity, compared to nanospheres, nanowires and nanodiscs, these branched nanostructures are expected to have a wide range of technological applications [135,137]. For example, they have interesting catalytic [114,115], magnetic [121,123,136], optical [115,135,136], and electronic [135,136] properties, and semiconductor heterostructures have potential uses in solar cells [136]. Branched nanostructures have also been suggested to be interconnections in the bottom-up self-assembly of future nanocircuits and nanodevices. Therefore, the mechanism by which these structures are formed [134,136] is a crucial step toward realizing synthetic control over their structural and functional properties. Synthetic control over the size and shape of these nanostructures is achieved using different chemical approaches. The controlled synthesis of these metal complex morphologies allows the modulation of the optical properties for each specific application.

To understand some optical behavior of metallic nanodendrites, we start from some consideration on the optical response of metal nanoparticles. Au and Ag nanoparticles show a characteristic absorption in the visible region of the electromagnetic spectrum due to the surface plasmon resonance. The position and width of the surface plasmon resonance band are considerably influenced by the size and shape of the nanocrystals. The wavelength dependence of the extinction coefficient is fairly well described by the Mie theory [62] for metal particles with sizes much smaller than the wavelength of light [127]. According to the Mie theory, the extinction coefficient is given by [138]

(1) $$k=\frac{18\pi N\;V\varepsilon_{m}^{3/2}}{\lambda }\frac{\varepsilon_{2}}{{\left[\varepsilon_{1}+2\varepsilon_{m}\right]}^{2}+\varepsilon_{2}^{2}}$$

where λ is the wavelength of incident light, and ε_1_ and ε_2_ are the real and imaginary parts of the complex dielectric function of the metal, and ε_m_ is the dielectric constant of the surrounding medium assumed to be an invariant with respect to the frequency of the light. The overall dielectric constant is given by

(2) $$\varepsilon =\varepsilon_{1}+i\varepsilon_{2}$$

**Figure 14:**
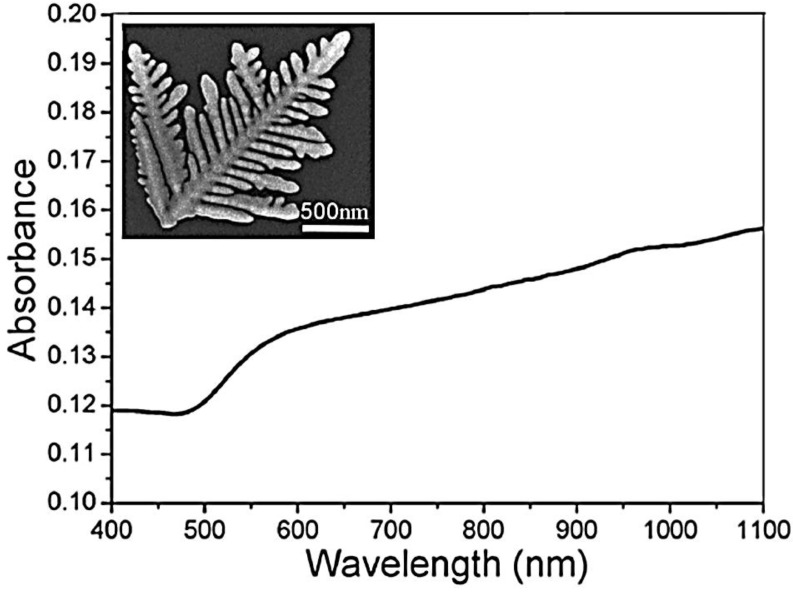
UV-vis absorption spectrum of Au nanodendrites. The inset shows the Au nanodendrites grown in mixed dodecyltrimethylammonium bromide/β-cyclodextrin solution. Reproduced with permission from [138]. Copyright American Chemical Society, 2010.

**Figure 15:**
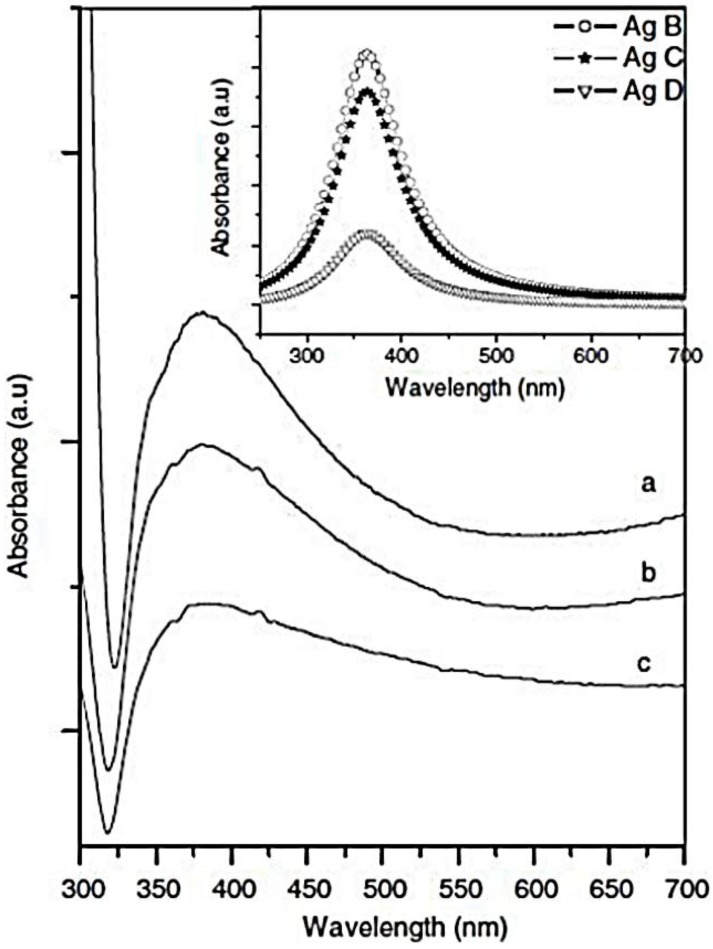
UV-vis absorption spectra of the samples: (**a**) Ag B, 0.3 M aqueous NH_3_ solution; (**b**) Ag C, 0.6 M aqueous NH_3_ solution and (**c**) Ag D, 1.2 M aqueous NH_3_ solution. The inset reports the simulated absorption spectrum from the Mie theory for the samples. Reproduced with permission from [139]. Copyright Institute of Physics, 2007.

One can readily see that the dependence of the extinction coefficient on wavelength is Lorentzian. Moreover, it is known that anisotropic metallic nanoparticles normally exhibit two principal SPR absorption peaks, characteristic of the short (transverse band) and long (longitudinal band) axes. To obtain more information about the changes in the optical response that occurs from a regular to a complex morphology of metallic nanostructures, we report here the results of some experimental works. Huang *et al.* [138] show the UV-vis absorption spectrum of the as-prepared Au nanodendrites suspended in water (see inset in Figure 14). The spectrum displays a gradual increase in absorption from ~500 nm to the near-IR region without indication of leveling off, which could be attributed to the longitudinal plasmon band, indicating a remarkable overlapping between the transverse band and the longitudinal band. It is well known that the position and intensity of the longitudinal band depend largely on the size, aspect ratio and mutual coupling of Au nanocrystals. The authors suggest that the observed overlapping between the transverse band and the longitudinal band for the obtained Au nanodendrites could be attributed to the polydispersity in the length and diameter of the trunks and branches, which may lead to a variety of sizes and aspect ratios. The multiple coupling between neighboring trunks and side branches may also result in a longitudinal plasmon band with the position lying in a wide range of wavelengths. The observed absorption spectrum may thus represent a contour combining the size, aspect ratio and coupling factors of the hierarchical dendritic structures. Conversely, Kaniyankandy *et al.* [139] report the optical properties of silver nanodendrites, synthesized by electrodeposition using AgNO_3_ as the source in ammoniacal solution. They report that SEM patterns of the samples show that as a consequence of the addition to the starting solution of a small amount of ammonia, the deposits (Ag B) show a dramatic change in morphology from irregularly shaped to long strands or rods. Furthermore, an increase of NH_3_ (Ag D) does not greatly affect the morphology. The structural characterizations, shown in this work, indicate that the average value of thickness of the central stem of a dendrite is ~50 nm. Along the central stem (with an average length of ~5 μm), branching is visible for every ~50 nm, and in some cases it is also hyperbranched. The branches of the dendrites are predominantly planar and hyperbranched. To illustrate the changes of the optical properties due to the different morphology of the resulting nanostructures, the authors compare the results of the simulation with the results of the optical measurements (Figure 15).

The simulation of the UV-vis spectra related to the metal colloids is shown in the inset of Figure 15. The authors highlight that the spectra show the Lorentzian nature of the band, and that the decrease in the size of nanoparticles leads to a decrease in the peak height and an increase in the peak width. The experimental UV-vis spectra (Figure 15) clearly give a single broad peak with maxima at λ = 380 nm. This indicates that the particles are predominantly spherical and do not have shape anisotropy. Additionally, the peak is highly asymmetric and has a large tail towards the lower energy region, *i.e.*, towards red. The peak extends to the entire visible region of the electromagnetic spectrum. This is contrary to the expected Lorentzian line shape. Kaniyankandy *et al.* explain that this result is due to the fact that, according to the Mie theory, metal nanoparticles are assumed to be non-interacting spheres. However, in their work, the metal nanoparticles are assembled in the form of a supramolecular dendritic structure. Therefore, a considerable amount of interaction is expected among the nanoparticles. Hence, the contribution from higher order multipoles and the distribution of depolarization factors have to be taken into consideration while simulating the absorption spectra. The distribution of depolarization factors in interacting metal spheres gives rise to a shape asymmetry for the SPR band. This has been previously observed by several authors [140]. The peak position at 380 nm is indicative of the particle sizes in the range 20–30 nm. Moreover, with an increase in the NH_3_ (hyperbranched nanostructures) content, the peak position shows only an extremely small shift towards the blue region, indicating a small decrease in the particle size, while the peak width increases with increasing ammonia content in the electrolyte. To explain these effects, the authors consider the size effects of metal nanoparticles. Depending on the size, there exist two regimes in the influence of particle size on the UV-vis spectrum [141]. The size regime is defined by the electron mean free path of the metal. For the bulk silver, the electron mean free path is ~52 nm. For particles smaller than the mean free path of the electrons, the influence of the grain boundary scattering cannot be ruled out, and must be taken into consideration. In the scenario when silver particle size is *>*52 nm, the peak position shows a red shift with increasing size, and the peak width increases with respect to the size (this is defined as an extrinsic size effect). For particle sizes lower than 52 nm, the peak position shows a very little change towards blue, and the peak width increases with decreasing size. Kaniyankandy *et al.* [138] indicate that this is the intrinsic size effect. In this case the size of all samples are in the range where the intrinsic size effect is operational. Therefore, the observation of the increase in the peak width with decreasing particle size can be explained on the basis of the intrinsic size effect. It is interesting to note that the SPR band spreads throughout the entire visible region of the electromagnetic spectrum. This observation is suggestive of aggregated silver nanoparticles, as well as in the present case where the aggregates form an ordered supramolecular structure. This hints at the applicability of these structures in SERS studies, because of an enhancement based on an electromagnetic mechanism, in which the high overlap of incident and scattered light with a metal SPR absorption band is important. The greater the overlap, the higher the enhancement. In the present case, since the SPR band extends throughout the entire visible region, the light of any wavelength in the visible region will be enhanced, thereby giving more flexibility with respect to the application in SERS. Furthermore, the highly branched dendritic structure has a higher surface area, which enables an increase in the sensitivity of SERS [142].

**Figure 16:**
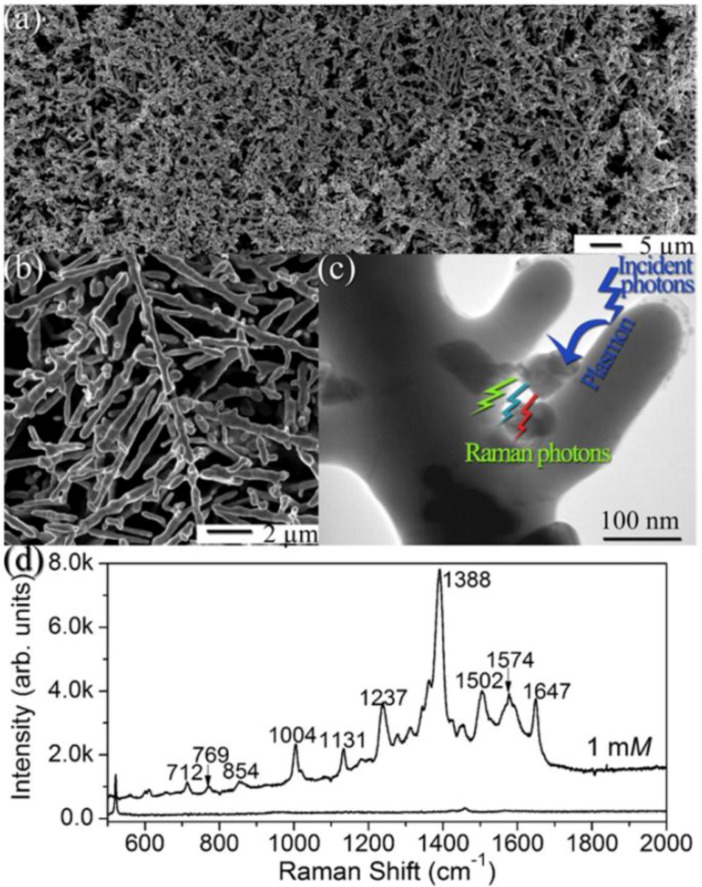
(**a**) Large-scale SEM image and (**b**) magnified image of silver nano-dendrites; (**c**) typical TEM image of C_60_ nanoclusters coupled with silver dendrite. The inset shows a schematic SERS process; light is coupled with a plasmon leading to an interaction and this is then Raman scattered by C_60_ nanoclusters on the surface of silver dendrite, and the outgoing plasmon is then scattered back into a photon; (**d**) SERS spectrum of C_60_ nanoclusters coupled with silver dendrites from a solution of 1 mM C_60_ in toluene. The Raman spectrum at the bottom corresponds to the sample of C_60_ nanoclusters coupled with Si wafer from 1 mM C_60_ toluene solution. Reproduced with permission from [143]. Copyright Institute of Physics, 2009.

To highlight the good response of the metallic nanodendrites as SERS-active substrates, we report the results of Qiu *et al.* [143]. The authors fabricated highly ordered and regular silver nanodendrites (on a silicon substrate) with tunable inter-nanowire gap size (Figure 16a,b) for placing analyte molecules (C_60_ nanoclusters) in hot-spots between closely spaced nanowires (Figure 16c), leading to tunable SERS enhancement. In order to give experimental evidence for the hot-spots of the form “metal/nanoclusters/metal,” the authors position many C_60_ nanoclusters as Raman probes in the junctions between the neighboring arms of silver nano-dendrites. Figure 16c shows the typical TEM image of C_60_ nanoclusters (the almost-circular regions between the two arms of the dendrite) coupled with silver nano-dendrite. C_60_ nanoclusters with sizes varying from 10 to 50 nm were clearly observed on the surface and between the arms of the dendrite, forming the “metal/nanoclusters/metal” structure. The SERS spectrum of such a system is reported in Figure 16d and it shows a variety of Raman bands (identified by the corresponding Raman shifts in Figure 16d). For comparison, the authors couple C_60_ nanoclusters on the silicon wafer, using a 1 mM C_60_ solution. No visible C_60_ Raman bands (see bottom of Figure 16d) are observed. The comparison, first of all, suggests that the strong enhancement for the dendritic pattern should be attributed to the fact that the nano-dendrites are assembled on the Si substrate with a very high density and with many horns and, thus, C_60_ nanoclusters in abundant hot-spots between closely spaced dendrite arms lead to intense SERS enhancement. The Raman spectrum of the C_60_ nanoclusters on the dendritic structures is analyzed by the authors supposing that the SERS process consists of a five-steps (as pictured in Figure 16d): (1) An ingoing photon interacts with the substrate exciting a plasmon; (2) The plasmon polarizes a molecule bound to the surface, creating a large effective dipole moment; (3) If the molecule now changes the vibrational state, then its molecular polarization alters; (4) The change in molecular polarization couples back into an emitted plasmon; (5) Finally, the plasmon couples with an outgoing Raman scattered photon. However, when the C_60_ nanoclusters are adsorbed on silver nano-dendrites, the symmetry of the C_60_ molecule is reduced, resulting in the splitting of the Raman bands from the degenerate modes of C_60_. As a result, the number of the vibration modes is greatly increased in the SERS spectrum (Figure 16d). A similar argument was invoked by Nikoobakht and El-Sayed [144] who forwarded that SERS intensities are far higher for molecules on aggregated gold nanorod deposits compared with monomeric nanorods, an observation attributed to an enhanced electromagnetic (EM) field in the inter-nanorod region. These studies indicate that the precise control of gaps between nanorods or nanowires on a SERS-active substrate is likely to be critical for the fabrication of substrates with uniformly high EFs, and for understanding collective surface plasmons. This is the critical reason many works carried out theoretical examinations of the local EM properties by the simulation method to assess the EM near-field distributions for a dendrite-like model pattern with different inter-arm gap dimensions.

The results of Qiu *et al.* therefore indicate that the nanodendritic architecture can be realized by multibranched metallic nanowires that act as efficient active SERS substrates. Regarding the one-dimensional nanostructure field, the next section aims to show that some interesting properties can be obtained from one-dimensional metal-based nanocomposite systems, demonstrating their potential as a basis for new-generation devices. The particular systems described aim to cross the localized surface plasmonic properties of metal nanoparticles with the waveguiding properties of silica nanowires.

## 6. Gold Nanoparticle-Embedded Dielectric Nanowires

Noble-metal nanoparticles encapsulated in a dielectric matrix have attracted sustained interest over several centuries owing to their unusual optical and electrical properties. Metal-dielectric nanocomposites show non-linear and fast optical response near the SPR frequency due to their enhanced third-order optical susceptibilities [145]. Thus, they have been applied in optical switching devices [146,147]. Several approaches, such as the sol-gel process, metal-dielectric co-sputtering deposition, metal-ion implantation into a dielectric matrix and pulsed laser deposition have been used to prepare metal-dielectric nanocomposites. In addition to the mentioned metal-dielectric nanocomposites that are mainly prepared in thin film or bulk forms, the one-dimensional noble-metal nanoparticle chain has become an intensive research focus because of its potential applications for nanoscale-integrated optics below the diffraction limit of light. It has been suggested theoretically and experimentally that the electromagnetic energy can be transported below the diffraction limit by a coupled-plasmon mode in the linear chains of noble-metal nanoparticles. The successful synthesis of a one-dimensional hybrid nanosystem known as a “peapod” offers a promising opportunity to realize a wide variety of functionalities. The best-known example among them is the all-carbon hybrid peapod structure, consisting of fullerene molecules encapsulated in single-walled carbon nanotubes, which has been demonstrated to show several unusual physical properties.

**Figure 17:**
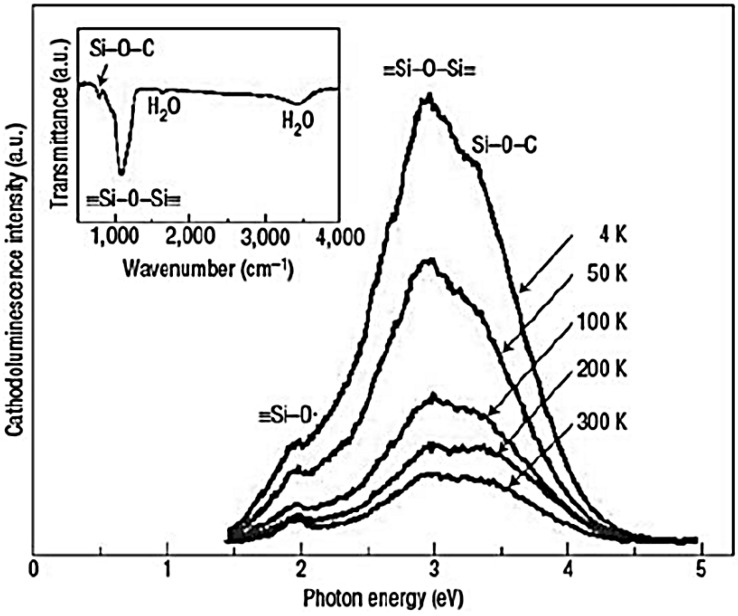
Temperature-dependent cathodoluminescent spectra of gold nano-peapodded silica nanowires. The inset shows the corresponding Fourier transform infrared spectroscopy (FTIR) spectrum. Reproduced with permission from [145]. Copyright Nature Publishing Group, 2006.

In the paper of Hu *et al.* [145], the approach for fabricating a self-organized metal nanoparticle chain encapsulated in a dielectric nanowire with SPR-induced conductivity is reported. Cathodoluminescence measurements on the gold nanopeapodded silica nanowires are carried out using a Gatan with an acceleration voltage of 8 keV. Figure 17 shows typical cathodoluminescent spectra of gold nanopeapodded silica nanowires at five different temperatures.

The authors highlight that the spectra are composed of three characteristic peaks at 1.9, 3.0, and 3.3 eV. The 1.9 eV band can be ascribed to the non-bridging oxygen hole centers or oxygen dangling bonds (≡Si–O). The 3.0 eV band corresponds to the two-fold coordinated Si lone pair centers originating from the formation of strain bonds (≡Si–O–Si≡), when mechanical stress is applied to the silica glass 22, 23. The 3.3 eV band may be correlated with the formation of the Si–O–C owing to the reaction between ≡Si–O defects and carbon atoms during nanowire growth, which has been reported in carbon-doped silica. As no carbon-containing gas reactants are intentionally introduced during nanowire growth, the authors suppose that the incorporated carbon might originate from the sputtering process of gold thin films under ambient air or the residual contaminant in their reaction chamber. Analyses of bonding configurations from FTIR data also confirm the existence of Si–O–C and ≡Si–O–Si≡ (Figure 17 inset).

**Figure 18:**
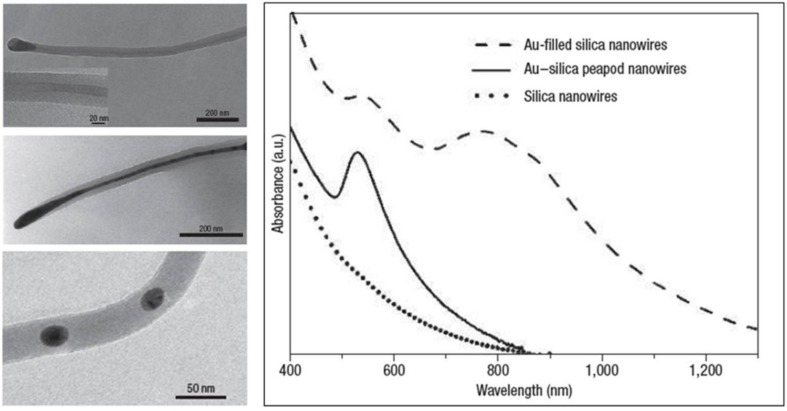
Optical absorption spectra for plain silica nanowires (dotted line), gold peapodded silica nanowires (solid line) and gold-filled silica nanowires wherein the aspect ratio of the gold segment is about 3–5 (dashed line). Reproduced with permission from [145]. Copyright Nature Publishing Group, 2006.

Ultraviolet-visible absorption measurements are performed on a set of the composite nanowires grown on quartz substrate with different gold filling in the silica nanowires. The room-temperature ultraviolet-visible absorption spectra for plain silica nanowires (dotted line), gold-peapodded silica nanowires (solid line) and gold-filled silica nanowires, wherein the gold segments show an aspect ratio of 3–5 (dashed line), are shown in Figure 18. The gold-filled and gold-peapodded silica nanowires reveal a pronounced SPR absorption band at 532 nm, whereas the plain silica nanowires do not show any SPR absorption. The SPR band at 532 nm is attributed to the transverse plasmon mode, and the longer wavelength band is associated with the longitudinal plasmon mode, which was observed for composite nanowires containing gold segments of high aspect ratio (see the dashed line in the plot in Figure 18). The absence of the longitudinal mode in the gold-peapodded nanowires (see the solid line in the plot in Figure 18) may be ascribed to the small aspect ratio (*<*1.2) of the gold nanoparticles. The observed background in the composite nanowires originates from an interband transition of valence electrons to the Fermi surface.

**Figure 19:**
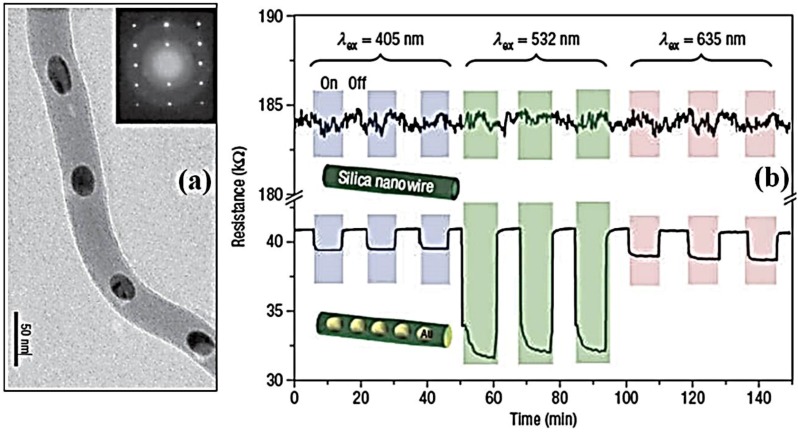
(**a**) TEM images showing the structure of metal nanoparticle-encapsulated silicon dioxide nanowire. The inset in in (**a**) shows an electron-diffraction pattern recorded along the [120] zone axis; (**b**) Photoresponse measurements. The room-temperature resistance response as a function of time to light illumination for plain silica nanowires (**upper** part) and gold nanopeapodded silica nanowires (**lower** part). Shaded (pink, excitation wavelength lex = 635 nm; green, lex = 532 nm; purple, lex = 405 nm) and unshaded regions mark the light-on and light-off periods. Reproduced with permission from [145]. Copyright Nature Publishing Group, 2006.

Photoresponse measurements based on the nanowire ensemble devices are conducted under alternate light illumination of different wavelengths and under dark conditions. The resistance of the hybrid peapod nanowire device shows nearly a five-fold smaller value than its counterpart without gold nanoparticles. More interestingly, the hybrid peapod nanowire device presents a wavelength-dependent photoresponse in resistance. The photoresponse behavior at an SPR wavelength of 532 nm shows a larger difference in resistance between the illumination on and off conditions (Figure 19).

The resistance change is nearly five times that observed using 632 and 405 nm light. Furthermore, an excitation-intensity-dependent resistance is measured using 532 nm light at various pumping powers. The authors believe that the enhanced photosensitive behavior observed from the hybrid nanowire device can be attributed to the SPR, as the hybrid nanowires have a pronounced SPR absorption at 532 nm. The enhancement of photoresponse behavior arising from the excitation of the surface plasmon has been reported for metal-oxide-metal (MOM) tunneling junctions, which is attributed to the generation of hot electrons owing to the decay of surface plasmon polaritons. The generated hot electrons drift or diffuse to the oxide barrier, and tunnel to the counter electrode. In the case of their study, one-dimensional gold nanopeapodded silica nanowire can be regarded as an MOM tunneling junction in series. The generation of hot electrons and electron tunneling through the oxide nanowires may contribute to the enhanced photoresponse behavior in our hybrid nanowire. The authors suggest that this simple one-step approach could be extended to the preparation of other metal-dielectric peapod-type nanowires with different functionalities.

**Figure 20:**
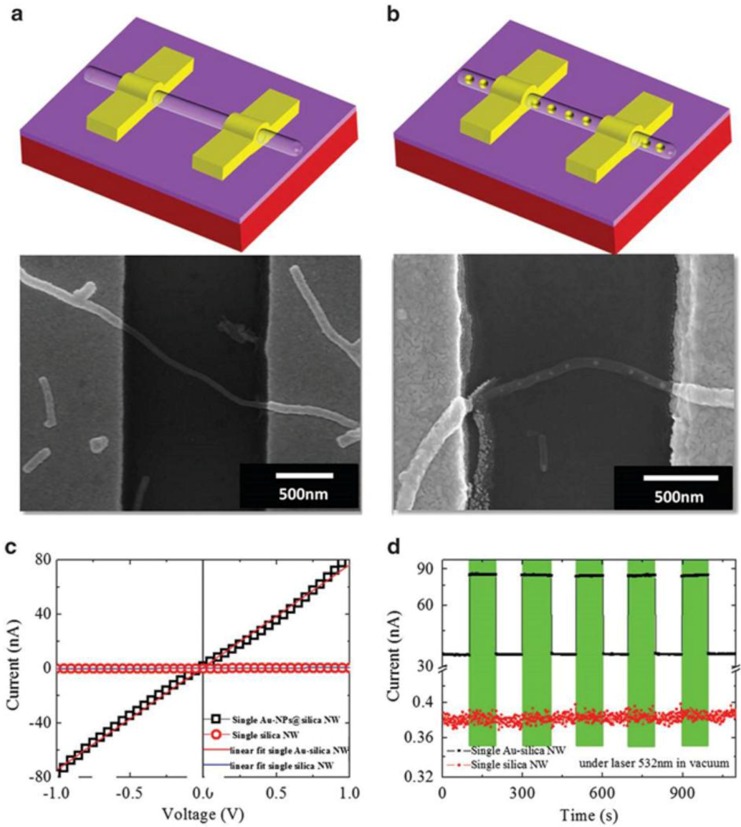
Field-emission scanning electron microscopy images of nanowire (NW) devices consisting of (**a**) plain silica NW (without the Au NPs) and (**b**) Au NPs-silica NW; (**c**) Dark I–V characteristics of single NW devices, with and without Au NP peapods, measured in vacuum, and the corresponding linear fit to the dataset; (**d**) Dark and photocurrent measured under vacuum in single NW devices, with and without Au NP peapods, as a function of time. The color bars indicate the duration of the 532 nm illumination. The measurement has been performed with a 1 V applied bias at room temperature. The bare silica NW showed no photoresponse, whereas the Au NPs-silica NW showed strong photoresponse. Reproduced with permission from [146]. Copyright Nature Publishing Group, 2013.

Furthermore, SiO_2_ nanowire (NW)-Au NPs composite systems are garnering great scientific and technological interest for their potential applications in, for example, electronic devices, biosensors, nanoscale optical devices and sensors. In particular, these pea-podded structures are used as light waveguides or biosensors, in line with the results reported by Wang *et al.* [146]. The authors report SPR-enhanced molecular oxygen sensing by single Au NPs-SiO*_x_* NW under 532 nm illumination (visible light) at room temperature. Figure 20a,b show field-emission scanning electron microscopy images of two terminal single-NW devices consisting of a silica NW without Au NPs (a) and a silica NW embedded with Au NPs (b). To understand the basic electrical and optoelectronic properties of the single-NW devices, the conventional current–voltage (I–V) and photoresponse were measured at room temperature in vacuum by the authors (Figure 20c,d). As shown in Figure 20c, the I–V plots of both NWs are linear, indicating the ohmic nature of the e-beam-defined contacts. The slope of the I–V plot indicates that the electrical conductivity of the Au NP-embedded silica NW is higher than that of the bare silica NW.

This trend occurs because the creation of more electron-hopping sites in the Au NPs-silica NW leads to enhanced conductivity [147]. During the growth of Au NP-peapodded silica NWs, residual Au atoms may partially incorporate into the silica matrix of the wire during the peapod formation. The incorporation of Au may produce impurities and/or defect-related hopping sites in the silica matrix [147]. The Au-embedded silica NW has higher conductivity than the plain silica NW, because of its shorter hopping length. The photoresponse measurements of the NW devices, conducted under alternating 532 nm illumination and dark conditions, are shown in Figure 20d. The plain silica NW device does not show any significant photoresponse; the ratio of photo and dark current is ~0.01. In contrast, the Au NPs-silica NW device presents a reversible photoresponse and a stable photocurrent when illuminated by 532 nm wavelength radiation (in Figure 20d the color bars indicate the duration of the 532 nm illumination), with a photo/dark current ratio of ~1.48. Assuming the band gap of silica NWs to be in the range of ~6.8 eV, and denying any absorption of the 532 nm light, these data suggest that the photocurrent can be attributed to the near-resonance SP absorption of the Au NPs (within the silica NWs) that has been reported at ~532 nm. So, under 532 nm illumination, the gold nanoparticles absorb photons so that electrons are (plasmonically) excited becoming charge carriers which can be transmitted through the silica nanowires by hopping processes between neighboring Au NPs. Taking into account these optoelectronic properties, such complex systems can be used as a light waveguide. Indeed, the resonant coupling between the incident light and the plasmonic resonances of Au NPs can induce an enhancement of the output signal.

## 7. Conclusions and Perspectives

In this paper, we reviewed the basic concepts related to some fabrication methods and the physico-chemical properties of complex-morphology metal nanostructures, focusing on the dependence of these properties on the specific nanostructure morphology. The main highlight of the review is that the fine wide-range control of the metal nanostructures’ morphological characteristics allow the wide-range tuning of their properties such as the electrical or optical ones. We tried to highlight that assemblies of complex-morphology metal nanostructures present intriguing properties, often more effective and versatile with respect to the properties of assemblies of spherical metal nanostructures. Therefore, in addition to chemical composition and size, the shape of the metal nanostructures is demonstrated to be a further fundamental parameter for reaching a wider range control of the properties of the systems towards functional technological applications in areas such as sensors, energy conversion, flexible electronics, optoelectronics, *etc*. Starting from the description of some peculiar cases (gold nanorings, gold nanorods, nanoporous gold structures, gold and silver nanodendrites, silica nanowires embedded with gold nanoparticles) we highlighted the disruptive role of the symmetry breaking in the metal nanostructure morphology in reaching unexplored nanomaterial behaviors. Due to the many unique characteristics of the complex-morphology metal nanostructures, we believe that the detailed understanding of the basic physical phenomena involved in the materials synthesis and electron transport and interaction with electromagnetic radiation can allow the desired control over properties and applications. The review thus emphasizes the basic microscopic mechanisms and processes and the general physical concepts suitable for the material properties’ interpretation and the structure-property correlations. Besides the basic processes and the general concepts, this review aims at a comprehensive schematization of the main technological applications currently in development somewhere in the world so as the future possible ones.

**Figure 21:**
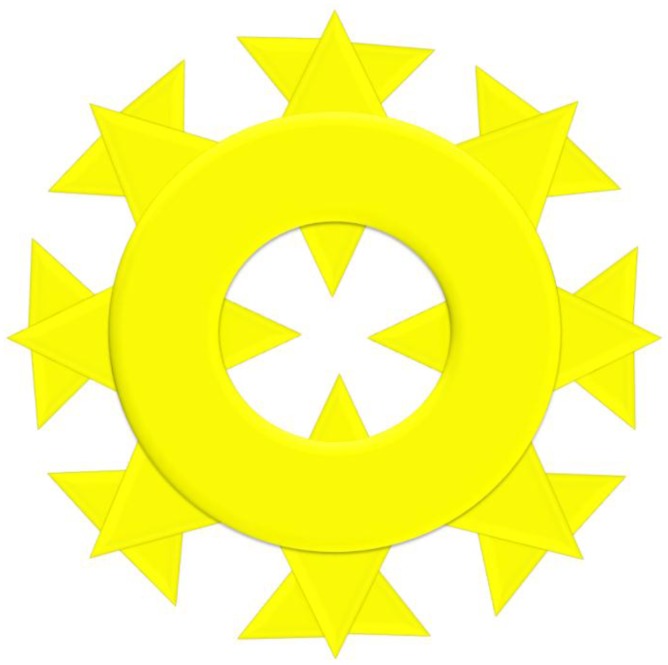
Picture of a possible complex-morphology Au nanostructure (plan-view) crossing the properties of Au nanorings with the properties of sharp Au tips typical of nanodendritic structures.

**Figure 22:**
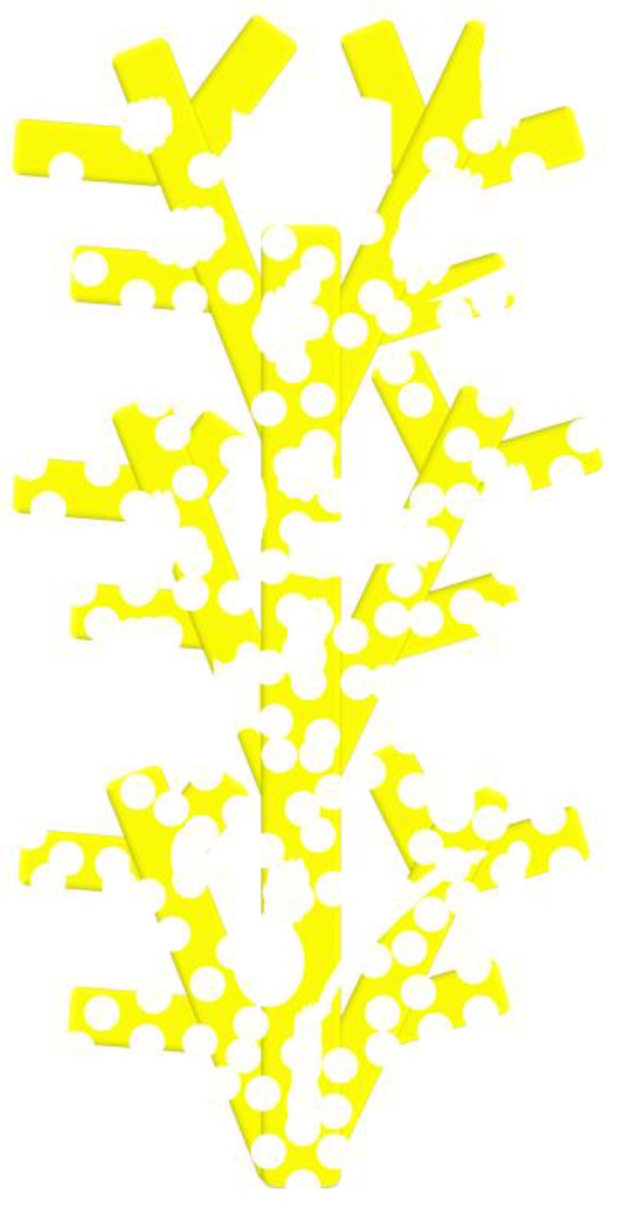
Picture of a possible nanoporous Au nanodendrites.

**Figure 23:**
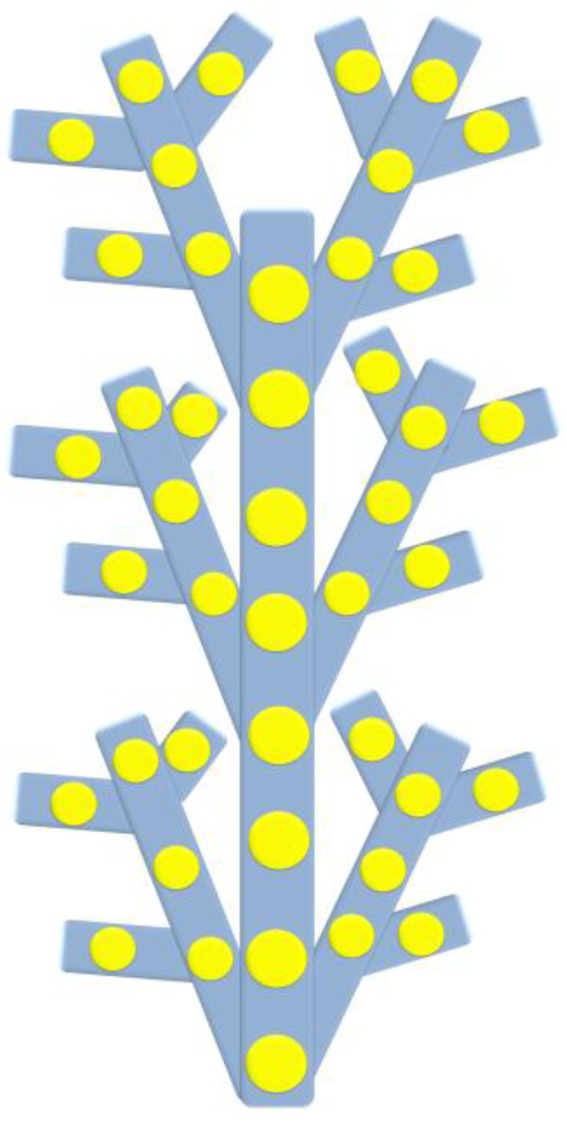
Picture of a possible branched silica nanowires embedded with Au nanoparticles.

Some perspectives can be drawn on the basis of the described systems and properties throughout the paper. In particular, we believe that the more interesting perspectives can be argued looking at the properties (and to the microscopic origin) of the illustrated complex-morphology metal-based nanostructures and thinking how these properties, presented singularly by the specific complex-morphology structure, can be crossed simultaneously in a unique nanostructure. Consequently, the prospective to realize some type of complex-morphology nanostructure originating from the complex-morphology characteristics of the two or more structures presented above aimed to cross the complex morphologies and meld the corresponding properties and performances. We briefly describe this idea through practical examples and propose the following:

(a) **Hybrid nanostructures***:* crossing the properties of Au nanorings and Au nanodendrities (see, for example, the possible architecture in Figure 21 (plan-view)). Possibly, such a type of structure could present LSPR frequency strongly dependent on the ratio of the thickness to ring outer radius and could simultaneously take advantage of the hot-spots for the electromagnetic regions originating in the inter-gap spaces between the sharp tips. So what can we expect from such a structure? Reasonably, very high surface-enhanced Raman scattering enhancement factors at a wavelength established by the ratio of the thickness to ring outer radius.
(b) **Nanoporous Au nanodendrites** (see the picture in Figure 22): crossing the properties of nanoporous Au and Au nanodendrites. In this case, the SERS effects characteristic of the nanodendritic Au structures (due to hot-spot engineering) could possibly be enormously enhanced thanks to the very exposed surface of the porous material where the analytes can connect and determine an increased sensitivity and efficiency.
(c) **Branched (*i.e.*, fractal-type) silica nanowires embedded with Au nanoparticles** (see Figure 23): In this case, we believe that properties of the silica nanowires embedded with Au nanoparticles, such as their photoconductivity, take advantage of the branched shape due to the occurrence of the hot-spots and, hence, of the consequent amplification of the electromagnetic field in between a larger number of in-gap regions between nanoparticles.

In addition to these fascinating complex-morphology metal-based nanostructures proposed as perspectives, nano-architectures to further improve efficiency with respect to electromagnetic-based phenomena, other possibilities include: porous gold nanoparticle- embedded silica nanowires, a system as pictured in Figure 21 but composed by nanoporous gold, multi-sharpened nanorices, *etc*. In general, the perspective idea can be: crossing specific morphologies in a unique structure (a truly hierarchically concept) to cross the properties and efficiencies (with respect to some specific process) to obtain, through mutual combination, un-precedented properties and higher efficiency with respect to the efficiencies of the original structures separately.

## Abbreviations

The following abbreviations are used in this manuscript:

EF	Enhancement factor
EM	ElectroMagnetic
FDTD	Finite Difference Time Domain method
FTIR	Fourier Transform Infrared Spectroscopy
LSPR	Localized Surface Plasmon Resonance
NP	NanoParticle
NPG	NanoPorous Gold
NW	NanoWire
SEM	Scanning Electron Microscopy
SERS	Surface-Enhanced Raman Scattering
SPP	Surface Plasmon Polariton
SPR	Surface Plasmon Resonance
TEM	Transmission Electron Microscopy
UV-vis	Ultraviolet-Visible
